# Inhibition of the MALT1-LPCAT3 axis protects cartilage degeneration and osteoarthritis

**DOI:** 10.1186/s12964-024-01547-4

**Published:** 2024-03-22

**Authors:** Vijay Kondreddy, Rajkumar Banerjee, B. L. A. Prabhavathi Devi, Kathirvel Muralidharan, Selvakumar Piramanayagam

**Affiliations:** 1 Department of Lipid Science and Technology, The Indian Institute of Chemical Technology, Uppal Road, Tarnaka, Hyderabad, 500007 India; 2 Division of Applied Biology, The Indian Institute of Chemical Technology, Tarnaka, Hyderabad, India

**Keywords:** LPCAT3, Lipid nanoparticles, MALT1, Cytokines, Eicosanoids, Osteoarthritis

## Abstract

The proinflammatory cytokines and arachidonic acid (AA)-derived eicosanoids play a key role in cartilage degeneration in osteoarthritis (OA). The lysophosphatidylcholine acyltransferase 3 (LPCAT3) preferentially incorporates AA into the membranes. Our recent studies showed that MALT1 [mucosa-associated lymphoid tissue lymphoma translocation protein 1]) plays a crucial role in propagating inflammatory signaling triggered by IL-1β and other inflammatory mediators in endothelial cells. The present study shows that LPCAT3 expression was up-regulated in both human and mice articular cartilage of OA, and correlated with severity of OA. The IL-1β-induces cell death via upregulation of LPCAT3, MMP3, ADAMTS5, and eicosanoids via MALT1. Gene silencing or pharmacological inhibition of LPCAT3 or MALT1 in chondrocytes and human cartilage explants notably suppressed the IL-1β-induced cartilage catabolism through inhibition of expression of MMP3, ADAMTS5, and also secretion of cytokines and eicosanoids. Mechanistically, overexpression of MALT1 in chondrocytes significantly upregulated the expression of LPCAT3 along with MMP3 and ADAMTS5 via c-Myc. Inhibition of c-Myc suppressed the IL-1β-MALT1-dependent upregulation of LPCAT3, MMP3 and ADAMTS5. Consistent with the in vitro data, pharmacological inhibition of MALT1 or gene silencing of LPCAT3 using siRNA-lipid nanoparticles suppressed the synovial articular cartilage erosion, pro-inflammatory cytokines, and eicosanoids such as PGE_2_, LTB_4_, and attenuated osteoarthritis induced by the destabilization of the medial meniscus in mice. Overall, our data reveal a previously unrecognized role of the MALT1-LPCAT3 axis in osteoarthritis. Targeting the MALT1-LPCAT3 pathway with MALT1 inhibitors or siRNA-liposomes of LPCAT3 may become an effective strategy to treat OA by suppressing eicosanoids, matrix-degrading enzymes, and proinflammatory cytokines.

**Graphical Abstract:**

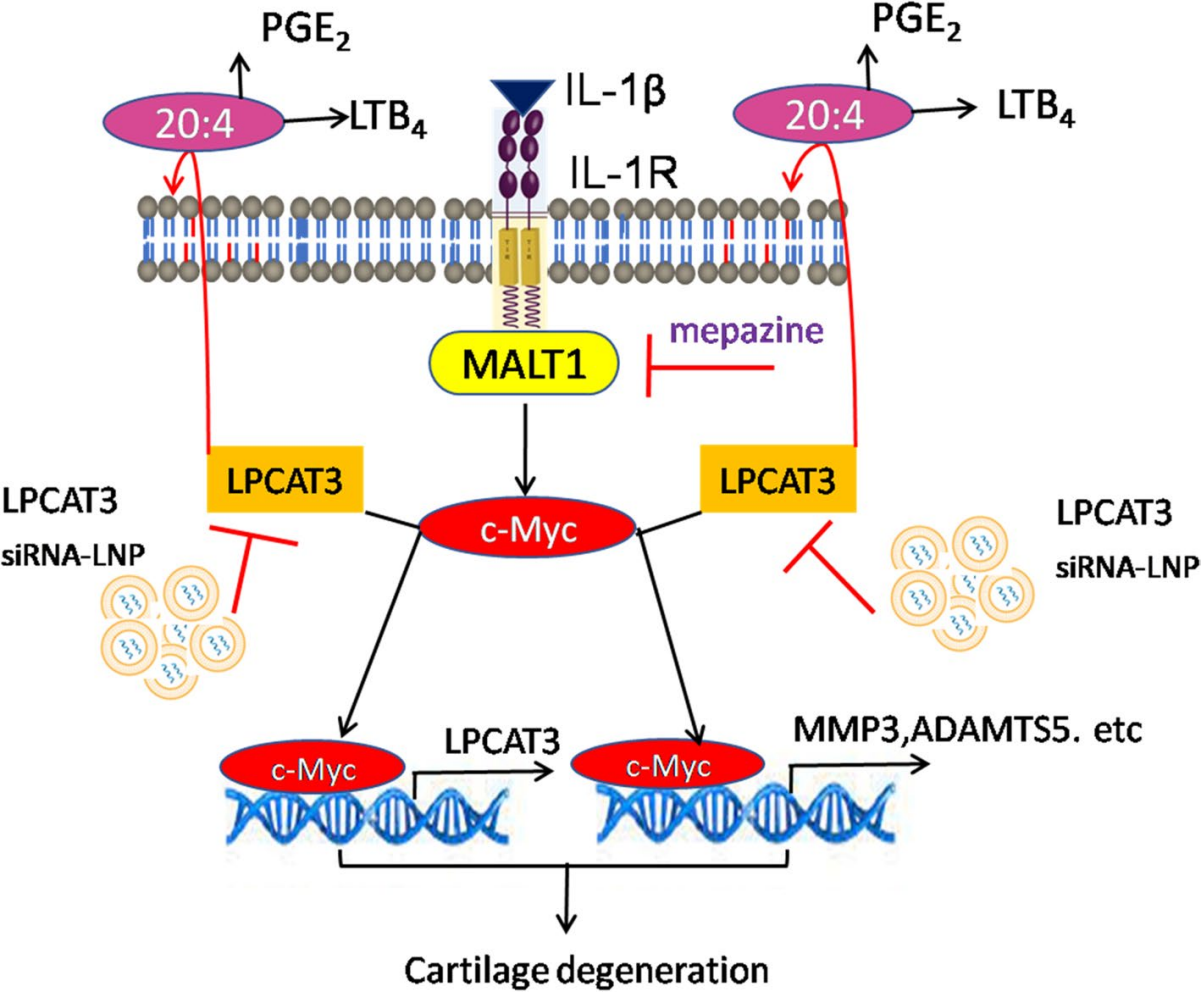

**Supplementary Information:**

The online version contains supplementary material available at 10.1186/s12964-024-01547-4.

## Introduction

Osteoarthritis (OA) is a chronic progressive synovial cartilage degenerative disease that affects over 500 million people globally [1–4]. There are no curative treatments that treat the degeneration of cartilage associated with OA [5]. The anti-inflammatory agents retard the progression of OA and hold promise for the development of novel, disease-modifying therapies [6, 7]. The arachidonic acid (AA) dependent metabolites namely prostaglandin E_2_ (PGE_2_) and leukotriene B_4_ (LTB_4_) play a crucial role in the regulation of pain, inflammation, and cartilage degeneration [3, 8]. The nonsteroidal anti-inflammatory drugs that target the arachidonic acid pathway have been exploited as potential therapies for OA. Hence, a precise understanding of the AA metabolism would lead to novel therapies for OA.

The salient feature of OA was the degradation of the cartilage matrix and suppression of extracellular matrix (ECM) proteins [9, 10]. The matrix metalloproteinases (MMPs) and ADAMTS5 degrade collagen type II and play an important role in the degeneration of cartilage [5, 9, 11]. Several lines of studies involving animals and humans suggest a critical role of IL-1β in the initiation and progression of OA [12–14]. The upregulation of IL-1β was correlated severity of OA [12, 14, 15]. The IL-1β induces proteinases such as MMPs and ADAMTS that culminate in cartilage degeneration [12, 14, 16]. Hence, inhibitors that block the action of IL-1β or overcome its signal transduction could be potential drugs to treat OA. The MALT1 is a critical regulator of cytokine and antigen receptor signaling, and MALT1 inhibitors attenuate the pathology of rheumatoid arthritis, ulcerative colitis, lymphomas, spondylitis, autoimmunity, multiple sclerosis, etc. [17–21]. Our recent studies have identified a novel pathway where the Gab2-MALT1 axis plays a critical role in the IL-1β-induced activation of NF-κB, expression of inflammatory genes, and vascular dysfunction [22, 23]. Currently, the role of IL-1β -MALT1 axis in the progression of OA remains elusive. Therefore, understanding the role of the IL-1β -MALT1pathway in cartilage dysfunction may lead to novel therapies that target MALT1 to treat OA.

Our previous studies have demonstrated that alteration of membrane AA, eicosapentaenoic acid (EPA), and docosahexaenoic acid (DHA) modifies the pathology of inflammatory bowel disease and cardiovascular diseases [24–27]. The AA which is released into the cytosol by the action of cPLA2 is utilized for the synthesis of PGE_2_ and LTB_4_ and other metabolites [28]. The consumed membrane pool of AA is replenished by LPCAT3 which preferentially incorporates AA into the membranes through acylation of lysoPC to phosphatidylcholine (PC) via Land’s cycle [29]. Growing evidence suggests that LPCAT3 is critically involved in the development of several pathologies that include skeletal muscle myopathy [30], atherosclerosis [31], acute kidney disease [32], nonalcoholic steatohepatitis [31], hyperuricemia [33], cancer [34], and other metabolic diseases. It has been reported that LPCAT3 was upregulated in OA and contributes to the ferroptosis of chondrocytes in osteoarthritis [35]. Currently, the precise role of LPCAT3 in the development and progression of OA remains known.

RNA interference (RNAi) that specifically silences gene expression mediated by small interfering RNA (siRNA) is a powerful tool for the treatment of various autoimmune disorders, viral infections and cancer [36]. The double-stranded siRNA with 21–25 nucleotides length, associates mainly with Dicer complex in the cytoplasm and cleaves target mRNA [36]. The siRNAs are mostly unstable in physiological fluids due to enzymatic degradation, limited membrane crossing, and challenge the delivery into the cytoplasm of target cells [37]. Thus, siRNA delivery systems have been developed which are mostly cationic liposomes to enhance the therapeutic efficacy of siRNA in clinical settings [38]. Previous studies reported that direct intra-articular injection of cytokine specific-siRNA using liposome -based delivery systems protect synovial cartilage in murine models of OA [39–41]. The treatment of rats with NF-κB (nuclear factor kappa-light-chain-enhancer of activated B cells) decoy oligodeoxynucleotides-loaded nanoliposomes led to an amelioration of arthritis [41]. These studies greatly support the use of siRNA-based therapies for the treatment of OA. Here, we designed a combination of LPCAT3 specific siRNA with nanocomplex and examined its potential for the treatment of OA. These formulations were intraarticularily injected in the DMM-induced OA mice, and therapeutic efficacy was evaluated in using histology studies, OARSI score and inflammatory markers.

Here, we found that the expression of LPCAT3 was upregulated in human and mice knee articular cartilage of OA and correlated with the severity of OA. We found that IL-1β upregulated the expression of LPCAT3 in chondrocytes besides MMP3 and ADAMTS5 via MALT1.The gene silencing of LPCAT3 in chondrocytes notably suppressed the secretion of eicosanoids and cytokines. The MALT1 knockdown using RNAi or chemical inhibition potentially reduced the IL-1β-induced catabolism of human cartilage explants and chondrocytes. We identified thatIL-1β-induces LPCAT3 via MALT1-c-Myc pathway. The Intraarticular (IA) administration of LPCAT3 siRNA- lipid nanoparticles or MALT1 inhibitor, mepazine, ameliorated OA in mice. This is the first study to describe the role of the MALT-LPCAT3 axis in the development of osteoarthritis.Our data supports that targeting the MALT1-LPCAT3 axis using MALT1 inhibitors or LPCAT3 siRNA-liposomes may become attractive therapies for the treatment of OA.

## Results

### LPCAT3 expression was upregulated in articular cartilage of human and mouse osteoarthritis

**Fig. 1:**
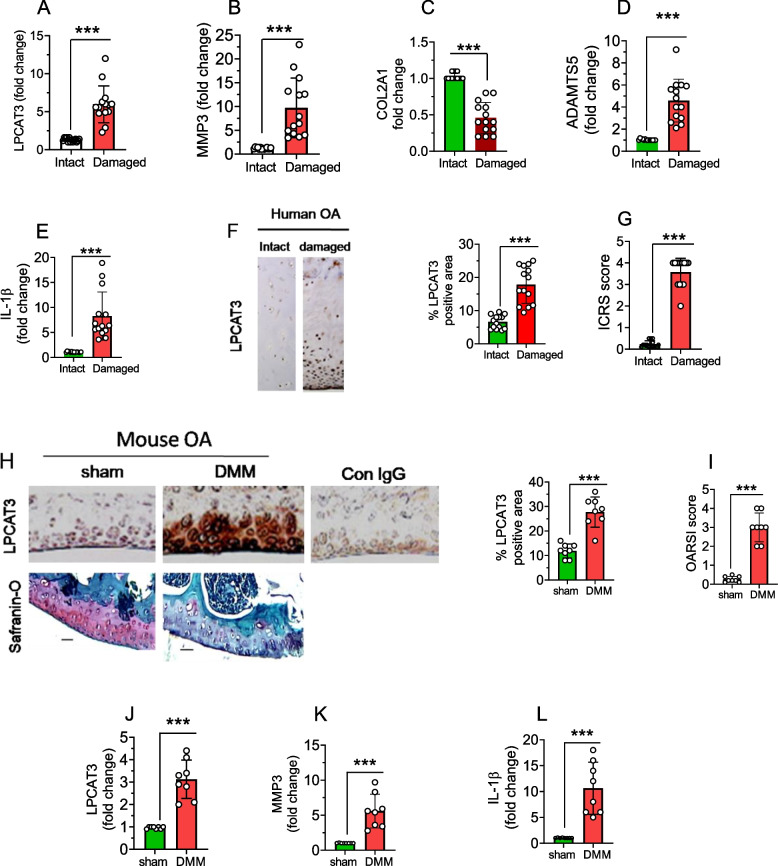
LPCAT3 expression was upregulated in human and mouse OA articular cartilage. **A**, **B**, **C**, **D**, **E** The mRNA expression levels of LPCAT3, MMP3, COL2A1, ADAMTS5, and IL-1β in non-involved (intact) and involved (damaged) regions of articular cartilage from OA patients (*n* = 14) were determined by qRT-PCR. Data are expressed as mean ± s.d. **p < 0.001 by Student’s two-tailed t-test. **F** Representative images of immunohistochemistry staining of LPCAT3 in intact and damaged regions of articular cartilage from human OA patients (*n* = 14). The % positive area of LPCAT3 in immunohistochemistry sections was quantified by Image J analysis (right). Data are expressed as mean ± s.d. ***p < 0.001 by Student’s two-tailed t-test. **G** The ICRS score corresponding to the images was evaluated. **H** The immunohistochemical staining (IHC) of articular cartilage sections with LPCAT3 and Safranin-O staining of sham-operated (*n* = 8) or DMM-induced OA (*n* = 8) mice (Scale bar- 50 µM). The % positive area of LPCAT3 in immunohistochemistry sections was quantified by Image J analysis (right). **I** The corresponding OARSI scores were evaluated. **J**, **K**, **L** The expression of LPCAT3, MMP3, and IL-1β were analyzed in mouse knee cartilage tissues by qRT-PCR. Data are expressed as mean ± s.d. **p < 0.01, ***p < 0.001 by Student’s two-tailed t-test

To determine the role of LPCAT3 in the pathogenesis of OA pathogenesis, we have collected cartilage tissues from OA patients undergoing knee replacement and examined the expression levels of LPCAT3 in the involved (damaged) and relatively uninvolved regions (intact) of cartilage. The gene expression analysis revealed that LPCAT3 expression was markedly upregulated in damaged regions compared with intact tissues. This was correlated with increased expression of MMP3, ADAMTS5, and IL-1β, and downregulation of COL2A1 (Fig. 1A, B, C, D, E). Consistent with this data, the immunostaining showed that LPCAT3 protein expression was notably increased in damaged OA cartilage compared to undamaged intact regions (Fig. 1F, G). In addition, we examined the expression of LPCAT3 in the articular cartilage of the OA mouse model induced by destabilization of the medial meniscus (DMM). Similarly, the LPCAT3 protein and mRNA expression was markedly elevated in the articular cartilage tissues of mice and correlated with disease activity at 8 weeks post-DMM surgery (Fig. 1H, I, J). Interestingly, this was associated with a prominent increase in the expression ofMMP3 and IL-1β (Fig. 1K, L).Convincingly; these results indicate that LPCAT3 expression was positively correlated with OA.

### IL-1β upregulates the expression of LPCAT3 in chondrocytes

**Fig. 2:**
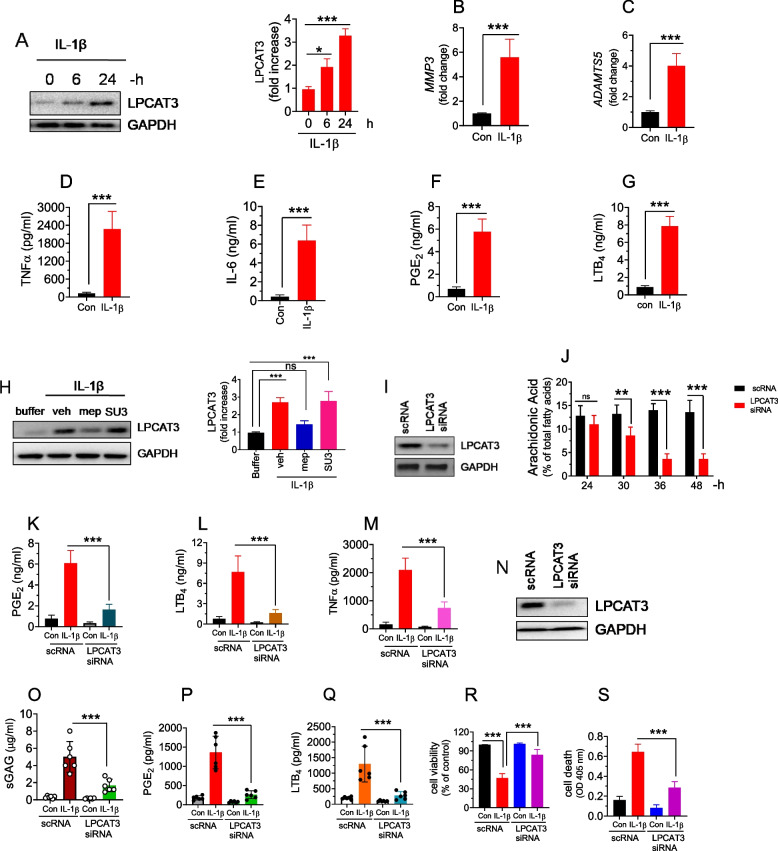
IL-1β induces LPCAT3 expression in human and mice chondrocytes. **A** The human chondrocytes were treated with IL-1β (10ng/ml) for 6-24h, the cell medium was collected and the cells were lysed in 2 × SDS sample buffer. The expression of LPCAT3 in the cell lysate was analyzed by immunoblotting using GAPDH as a loading control. Densitometric analysis of immunoblots was performed and the data was presented on the right side. Data are shown as mean ± SD of 3 independent experiments. *p < 0.05; ***p < 0.001. **B**, **C** The total RNA was isolated from the IL-1β-stimulated chondrocytes (24 h) and expression of *MMP3* (**B**) and *ADAMTS5* (**C**) mRNA was analyzed by RT-PCR. **D**, **E**, **F**, **G** The IL-1β-induced (24 h) secretion of cytokines TNFα (**D**), IL-6 (**E**), and eicosanoids, PGE_2_ (**F**) and LTB_4_ (**G**) in the medium were analyzed by ELISA, ***p < 0.001. **H** The human chondrocytes were pretreated with vehicle, mepazine (10 μM), or SU3327 (10 μM) for 1h, and the cells were stimulated with IL-1β for 24 h. The cells were lysed in 2 × SDS sample buffer and expression of LPCAT3 in the cell lysate was analyzed by immunoblotting. Densitometric analysis of immunoblots was performed and the data was presented on the right side. Data are shown as mean ± SD of 4 independent experiments. ***p < 0.001; ns: not significant. **I** The human chondrocytes were transfected with 200 nM of scrambled RNA (scRNA) or LPCAT3 siRNA. 48 h post-transfection, the cells were lysed in 2 × SDS-sample buffer, the proteins were separated by SDS-PAGE and the efficiency of knockdown was analyzed by immunoblotting. The LPCAT3-specific siRNA transfection inhibited the expression of LPCAT3 by 85% in chondrocytes. **J** The cells were lysed at different time intervals as shown in the figure and total lipids were isolated. The fatty acid methyl esters (FAME) were analyzed by gas chromatography. The arachidonic acid content in the cells was presented as a percent of total fatty acids. Data are shown as mean ± SD of 3 independent experiments. Ns- not significant; *p < 0.05; **p < 0.01; ***p < 0.001. **K**, **L**, **M** The LPCAT3 silenced chondrocytes were treated with IL-1β for 24 h and the cell medium was collected. The eicosanoids, PGE_2_ (**K**), LTB_4_ (**L**), and TNFα (**M**) were analyzed in the medium by ELISA. Data are shown as mean ± SD of 4 independent experiments. ***p < 0.001. **N** The human cartilage explants were transfected with 200 nM of scrambled RNA (scRNA) or LPCAT3 siRNA. 48 h post-transfection, the cells were lysed in 2 × SDS-sample buffer, the proteins were separated by SDS-PAGE and the efficiency of knockdown was analyzed by immunoblotting. **O**, **P**, **Q** The LPCAT3 silenced human cartilage explants were treated with IL-1β for 24 h and the cell medium was collected. The sGAG in the medium was analyzed by DMMB assay (**O**), and The PGE_2_ (**P**) and LTB_4_ (**Q**) were analyzed in the medium by ELISA. **R**, **S** The human chondrocytes were transfected with 200 nM of scrambled RNA (scRNA) or LPCAT3 siRNA. 48 h post-transfection, cells were treated with IL-1β for 24 h, and cell viability (MTT assay) (**R**), and cell death were analyzed by ELISA (**S**). Data are shown as mean ± SD of 3 independent experiments. ***p < 0.001

The human cartilage sections show that the upregulation of LPCAT3 was associated with a prominent increase in the expression of IL-1β and matrix-degrading enzymes. Therefore, we have examined whether IL-1β induces the expression of LPCAT3 in chondrocytes. Immunoblotting analysis showed thatIL-1β treatment for 6–24 h significantly induced the expression of LPCAT3 protein in human chondrocytes (Fig. 2A). This was associated with increased expression of MMP3, ADAMTS5, and secretion of TNFα, IL-6, PGE_2,_ and LTB_4_ (Fig. 2B, C, D, E, F, G). The IL-1β induces gene expression through the activation of various MAP kinases such as ERK, JNK, and P38. Our recent studies revealed that MALT1 mediates the IL-1β-induced signal transduction in endothelial cells. To determine whether MALT1 or MAPK were involved in the IL-1β-induced LPCAT3 expression, we have pretreated chondrocytes with specific inhibitors of MALT1 (mepazine) or JNK (SU3327). We found that MALT1 inhibition significantly reduced the IL-1β-induced expression of LPCAT3 whereas JNK inhibition does not affect the expression (Fig. 2H). This data suggests that IL-1β-induces LPCAT3 via MALT1.

The IL-1β upregulated the expression of LPCAT3 and enhanced the secretion of cytokines and arachidonic acid-derived eicosanoids (PGE_2_ and LTB_4_) in chondrocytes. The increased secretion of eicosanoids might be due to the increased incorporation and availability of substrate, arachidonic acid. To confirm this, we have knockdown LPCAT3 using LPCAT3-specific siRNA in chondrocytes. Immunoblotting analysis showed that LPCAT3 siRNA inhibited the expression of LPCAT3 protein by around 85% compared to scramble RNA (Fig. 2I). We have analyzed the arachidonic acid content in chondrocytes using gas chromatography at different time intervals post-siRNA transfection. We found that the arachidonic acid content was unaffected at 24 h post-transfection of LPCAT3 siRNA. However, the AA levels were reduced by 80% at 36–48 h post-transfection (Fig. 2J). Most importantly, LPCAT3 inhibition by RNAi markedly suppressed the IL-1β-induced secretion of PGE_2_, LTB_4_, and TNFα (Fig. 2K, L, M). This data confirms that LPCAT3 depletion suppresses AA levels in chondrocytes, and inhibits the synthesis of eicosanoids. Next, to assess the role of LPCAT3 in cartilage catabolism in intact human cartilage explants, we have silenced LPCAT3 using RNAi technology. We found that there was 90% inhibition in the expression of LPCAT3 in the cartilage tissues treated with LPCAT3 siRNA compared to scrambled RNA (Fig. 2N). The IL-1β stimulation significantly increased the cartilage catabolism as evidenced by a notable increase in the sulfated GAGs in the cell medium (Fig. 2O, P, Q). This was associated with increased secretion of PGE_2_ and LTB_4_. However, sulfated GAGs were significantly lower in the LPCAT3 siRNA-treated cartilage explants compared to the siRNA group. The LPCAT3 siRNA significantly lowered the PGE_2_ and LTB_4,_ and TNFα secretion in cartilage explants (Fig. 2O, P, and Q). In agreement with these results, IL-1β significantly reduced cell viability by inducing cell death in chondrocytes. In contrast, LPCAT3 gene silencing significantly protected the cell viability through inhibition of IL-1β-induced cell death (Fig. 2R, S).This compelling evidence suggests that the upregulation of LPCAT3 in human cartilage tissues promotes cartilage catabolism through increased arachidonic substrate availability and generation of eicosanoids and cytokines.

### Role of MALT1 in the IL-1β-induced cartilage degeneration

**Fig. 3:**
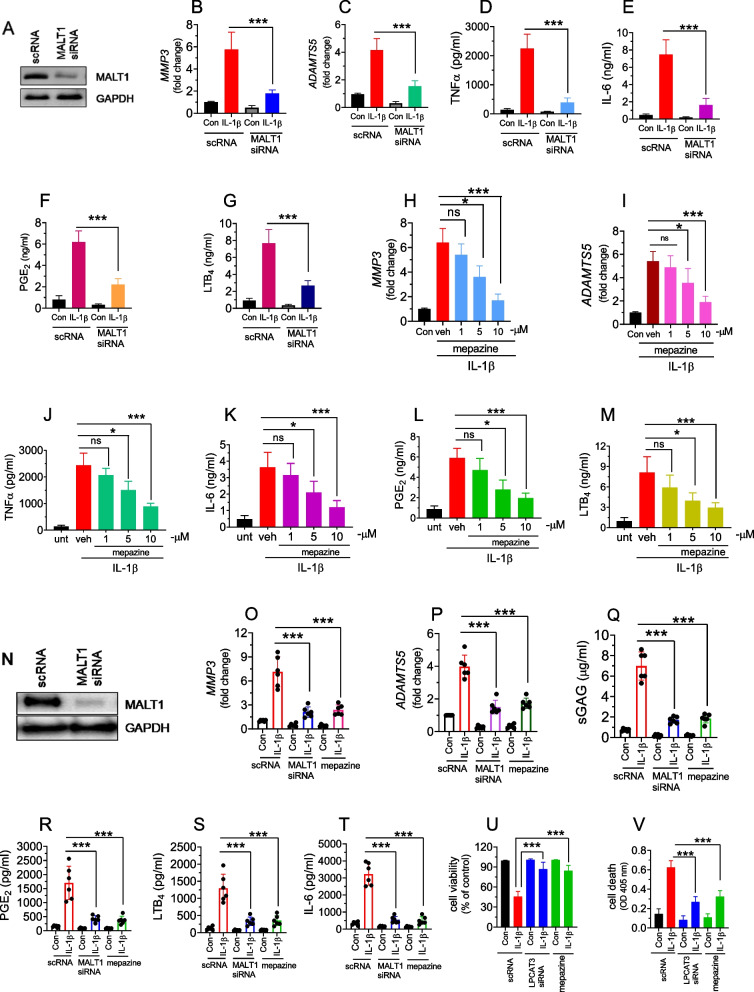
Gene silencing or pharmacological inhibition of MALT1 suppresses IL-1β-induced chondrocyte degeneration. **A** The human chondrocytes were transfected with 200 nM of scrambled RNA (scRNA) or MALT1 siRNA. 48 h post-transfection, the cells were lysed in 2 × SDS-sample buffer, the proteins were separated by SDS-PAGE and the efficiency of knockdown was analyzed by immunoblotting. **B**, **C** The MALT1 silenced chondrocytes were treated with IL-1β for 24 h and the cell medium was collected. The total RNA was isolated from the cells and the expression of *MMP3* (**B**) and *ADAMTS5* (**C**) mRNA was analyzed by RT-PCR. The TNFα (**D**), IL-6 (E), PGE_2_ (**F**), and LTB_4_ (**G**) levels were analyzed in the medium by ELISA. Data are shown as mean ± SD of 3 independent experiments. ***p < 0.001. **H** The human chondrocytes were pretreated with vehicle, mepazine (1–10 μM) for 1h, and the cells were stimulated with IL-1β for 24 h. The total RNA was isolated from the cells and the expression of *MMP3* (**H**) and *ADAMTS5* (**I**) mRNA was analyzed by RT-PCR. The TNFα (**J**), IL-6 (**K**), PGE_2_ (**L**), and LTB_4_ (**M**) levels were analyzed in the medium by ELISA. **N** The human cartilage explants were transfected with 200 nM of scrambled RNA (scRNA) or MALT1 siRNA. 48 h post-transfection, the cells were lysed in 2 × SDS-sample buffer, the proteins were separated by SDS-PAGE and the efficiency of knockdown was analyzed by immunoblotting. **O**, **P** The MALT1 silenced human cartilage explants were treated with IL-1β for 24 h and the cell medium was collected. The total RNA was isolated from the cells and the expression of *MMP3* (**O**) and *ADAMTS5* (**P**) mRNA was analyzed by RT-PCR. **Q** The sGAG in the medium was analyzed by DMMB assay. The PGE_2_ (**R**), LTB_4_ (**S**), and IL-6 (**T**) levels in the medium were analyzed by ELISA. **U**, **V** The human chondrocytes were transfected with 200 nM of scrambled RNA (scRNA) or MALT1 siRNA. 48 h post-transfection, cells were treated with IL-1β for 24 h, and cell viability (MTT assay) (**U**), and cell death was analyzed by ELISA (**V**). In the mepazine group, mepazine (10μM) or vehicle was added 1 h prior to IL-1β treatment. Data are shown as mean ± SD of 3 independent experiments. ***p < 0.001

Our recent studies showed that the Gab2-MALT1 axis propagates signal transduction of various proinflammatory cytokines including IL-1β, and thrombin in endothelial cells [22, 23]. In the current study, inhibition of MALT1 markedly lowered the expression of LPCAT3. To elaborate on the role of MALT1 in cartilage degeneration, we have specifically silenced MALT1 in chondrocytes. Immunoblotting analysis showed that MALT1 siRNA inhibited the expression of MALT1 protein by around 80% compared to scramble RNA (Fig. 3A). Interestingly, inhibition of MLAT1 by RNAi notably suppressed the expression of MMP3, ADAMTS5, and secretion of TNFα, IL-6, PGE_2,_ and LTB_4_ (Fig. 3B, C, D, E, F, G). In addition, pharmacological inhibition of MALT1 by mepazine dose-dependently suppressed the expression of MMP3, ADAMTS5, and secretion of TNFα, IL-6, PGE_2,_ and LTB_4_ (Fig. 3H, I, J, K, L, M).

Next, to assess the role of MALT1 in cartilage catabolism in human cartilage explants, we have silenced MALT1 using RNAi. Human cartilage tissues manifest marked expression of MALT1. There was around 90% inhibition in the expression of MALT1protein in cartilage tissues treated with MALT1 siRNA compared to scramble RNA (Fig. 3N). Similar to isolated chondrocytes, IL-1β significantly increased the expression of MMP3 and ADAMTS5 in human cartilage explants (Fig. 3O, P).This was associated with enhanced secretion of sulfated GAGs, PGE_2_ and LTB_4,_ and IL-6 in the cell medium (Fig. 3Q, R, S, T). However, sulfated GAGs were significantly lower in the MALT1 siRNA-treated cartilage explants compared to the scRNA group. The MALT1 siRNA significantly lowered the PGE_2_ and LTB_4,_ and IL-6 secretion in cartilage explants (Fig. 3Q, R, S, T). Most importantly, MALT1 gene silencing or chemical inhibition by mepazine significantly protected the cell viability through inhibition of IL-1β-induced cell death (Fig. 3U, V). Overall, these results convincingly demonstrate the critical role of MALT1 in the IL-1β -induced cartilage degeneration.

### IL-1β-induces LPCAT3 via MALT1- C-Myc pathway in chondrocytes

**Fig. 4:**
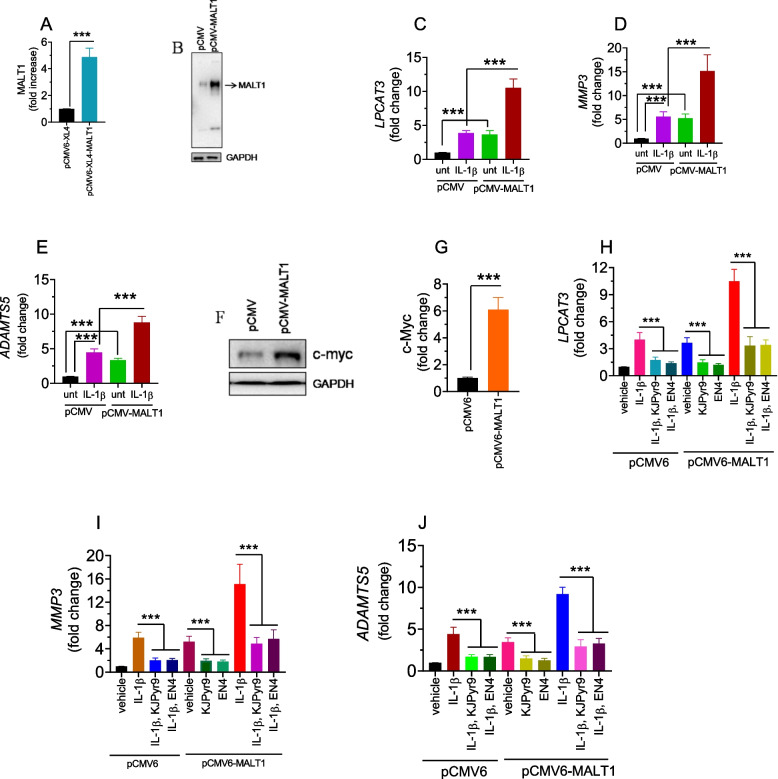
IL-1β-induces LPCAT3 via MALT1-c-Myc pathway in chondrocytes. **A**, **B** The human chondrocytes were transfected with pCMV-XL4-MALT1 plasmid or empty plasmid (pCMV).48 h post-transfection, the total RNA was isolated and the expression of MALT1 was analyzed by RT-PCR (**A**). Alternatively, the cells were lysed in 2 × SDS-sample buffer, the proteins were separated by SDS-PAGE and the expression of MALT1 was analyzed by immunoblotting (**B**). **C**, **D**, **E** The human chondrocytes were transfected with pCMV-XL4-MALT1 plasmid or empty plasmid (pCMV).48 h post-transfection, the cells were treated with IL-1β for 24 h. Later, the total RNA was isolated and the expression of *LPCAT3* (**C**), *MMP3* (**D**), and *ADAMTS5* (**E**) were analyzed by RT-PCR. Data are shown as mean ± SD of 3 independent experiments. ***p < 0.001. **F**, **G** The human chondrocytes were transfected with pCMV-XL4-MALT1 plasmid or empty plasmid (pCMV).48 h post-transfection, the cells were lysed in 2 × SDS-sample buffer, the proteins were separated by SDS-PAGE and the expression of c-Myc was analyzed by immunoblotting (**F**). Alternatively, total RNA was isolated and the expression of c-Myc was analyzed by RT-PCR (**G**). **H**, **I**, **J** The human chondrocytes were transfected with pCMV-XL4-MALT1 plasmid or empty plasmid (pCMV).48 h post-transfection, the cells were pretreated with c-Myc inhibitors, KJPyr9 (5 μM) or EN4, 5 μM for 1h. Later, the cells were stimulated with IL-1β for 24h. The total RNA was isolated and the expression of *LPCAT3* (**H**), *MMP3* (**I**), and *ADAMTS5* (**J**) were analyzed by RT-PCR. Data are shown as mean ± SD of 3 independent experiments. ***p < 0.001

Our gene silencing or pharmacological inhibition studies confirm the involvement of LPCAT3 and MALT1 in IL-1β-induced cartilage degeneration. To establish the molecular links between the MALT1-LPCAT3 axis, we have overexpressed MALT1 in chondrocytes using a pCMV-XL4-MALT1 plasmid construct (Origene, MD, USA). The RT-PCR and immunoblotting analysis confirmed that MALT1 mRNA and protein were notably elevated in chondrocytes incubated with pCMV-MALT1 plasmid compared to empty vector control (Fig. 4A, B). Interestingly, MALT1 overexpression significantly upregulated the expression of LPCAT3, MMP3, and ADAMTS5 in chondrocytes compared to vector control cells (Fig. 4C, D, E). This expression of LPCAT3, MMP3, and ADAMTS5 were heightened by IL-1β treatment (Fig. 4C, D, E). These results were in harmony with our gene silencing studies of MALT1 and potentially support that MALT1 mediates IL-1β-induced expression of matrix-degrading enzymes in chondrocytes. Several studies demonstrate that c-Myc mediates the IL-1β-induced gene signature [42–44]. Moreover, c-Myc was actively involved in the molecular events that underlie IL-1β-induced cartilage degeneration in OA [44, 45]. Therefore, we hypothesized whether c-Myc orchestrates the activation of the IL-1β-MALT1-LPCAT3 axis in chondrocytes. Supporting this hypothesis, we found that there was a significant accumulation of c-Myc mRNA and protein in the MALT1 overexpressed chondrocytes (Fig. 4F, G). Most importantly, inhibition of c-Myc by small molecules that interfere with c-Myc target gene expression (KJPyr9, EN4), markedly suppressed the expression of LPCAT3, MMP3, and ADAMTS5 in IL-1β treated and/or MALT1 overexpressed chondrocytes (Fig. 4H, I, J). These data clearly suggest that the MALT1-c-Myc pathway regulates the IL-1β-induced cartilage catabolic gene signature in chondrocytes.

### Pharmacological inhibition of MALT1 dampens inflammation, and loss of cartilage, and attenuates OA in mice

**Fig. 5:**
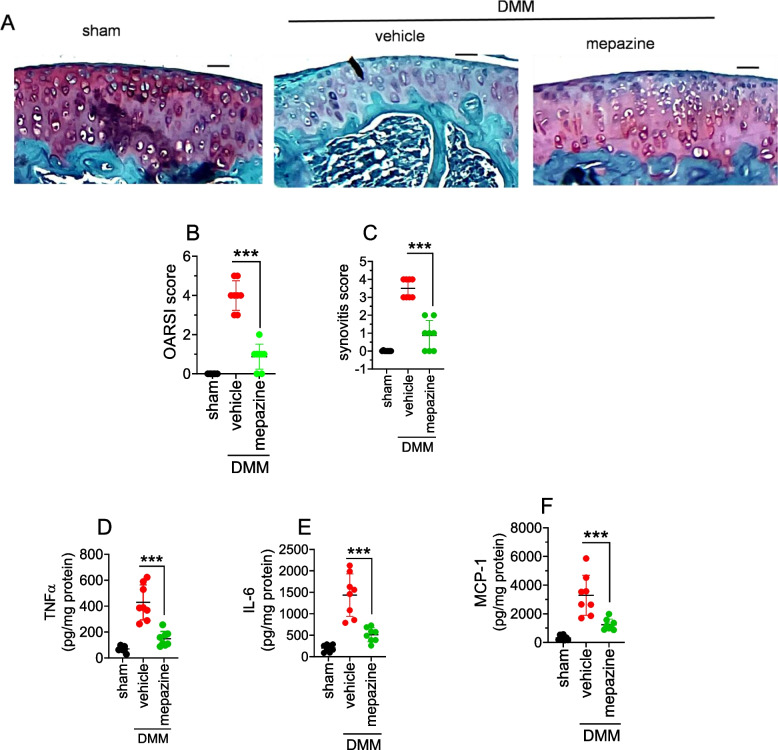
Intraarticular administration of mepazine protects osteoarthritis in mice. **A** OA was induced in knee tissues of mice by performing DMM surgery in 10–12-week-old C57WT mice, followed by intraarticular injection of mepazine (*n* = 8 mice per group), administered immediately and thereafter weekly twice post-DMM surgery at a dosage of 10 mg/kg body weight of mice in 20 μl volume. The knee tissues were collected at 8 weeks post DMM and they were fixed in formalin or frozen. The formalin-fixed sections were decalcified and stained with Safranin O fast green. The images represent data from 8 mice per group (Scale bar- 50 µM). **B**, **C** The sections were graded by 2 blinded observers according to OARSI guidelines and the OARSI score (**B**), and synovitis score (**C**) were presented. Data represent mean ± SD of 3 independent experiments, a total of 8 animals in a group. ***P < .001. **D**-**H** The frozen cartilage tissues were homogenized in the RIPA buffer containing protease inhibitors and indomethacin (1 mg/mL), and centrifuged at 10,000*g* at 4°C to remove tissue debris. The supernatants were collected. The cytokines such as TNFα (**D**), IL-6 (**E**), and MCP-1 (**F**), and eicosanoids PGE_2_ (**G**) and LTB_4_ (**H**) were analyzed by ELISA. Data represent mean ± SD of 2 independent experiments, a total of 8 animals in a group. ***P < .001

Our in vitro studies on chondrocytes and cartilage explants suggest that MALT1 was critically involved in IL-1β-induced expression of matrix-degrading enzymes, eicosanoids, and cytokines that collectively regulate cartilage destruction. The MALT1 inhibition using mepazine attenuates cartilage degeneration by interfering with IL-1β-induced chondrocyte damage. To evaluate whether MALT1 inhibition exhibits cartilage protective benefits in vivo, we have induced OA in mice using DMM-surgery and administered mepazine weekly twice for 8 weeks. The safranin staining images of knee tissues of vehicle-treated mice that underwent DMM surgery manifest critical loss of articular cartilage compared to the sham group (Fig. 5A). The OARSI and synovitis scores confirmed these results (Fig. 5B, C & Supplementary Fig. 2A). This was associated with significant upregulation of cytokines (TNFα, IL-6, and MCP1) and arachidonic acid-derived eicosanoids (PGE_2_ and LTB_4_) (Fig. 5D, E, F, G, H). In contrast, mepazine treatment significantly conserved the loss of synovial cartilage tissue compared to vehicle treated group. This was associated with a notable reduction in TNFα, IL-6 MCP1, and eicosanoids such as PGE_2_ and LTB_4_. Hence, MALT1 inhibition suppresses inflammation and attenuates OA in mice (Fig. 5D, E, F, G, H).

### The LPCAT3 gene knockdown mitigates DMM-induced inflammation and cartilage degeneration in mice

**Fig. 6:**
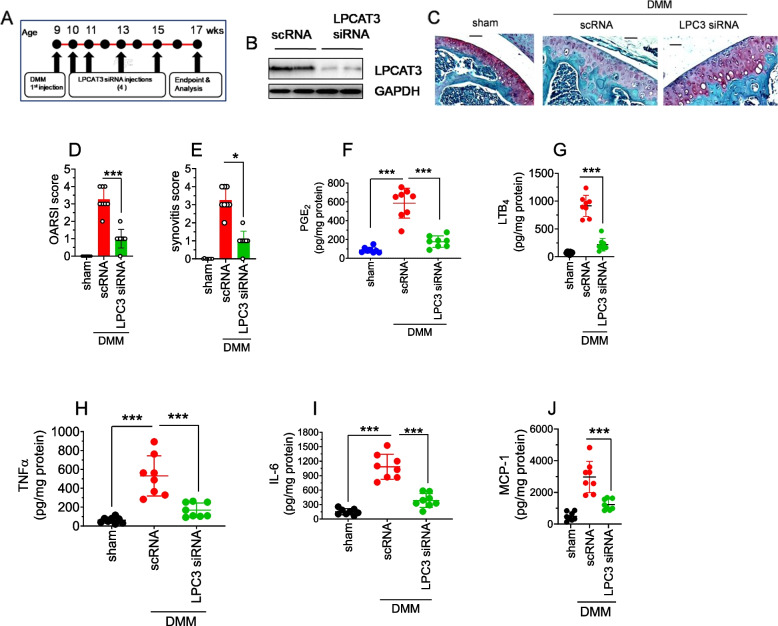
Intraarticular administration of LPCAT3-siRNA using lipid nanoparticles ameliorates OA in mice. **A** OA was induced in knee tissues of mice by performing DMM surgery in 10-12 week-old C57WT mice, followed by intraarticular injection of LPCAT3 siRNA lipid nanoparticles (LPC3 siRNA) or scrambled siRNA (siRNA) or PBS as untreated control (sham) (*n* = 12 mice per group), administered immediately and thereafter at 1, 2, 4, and 6 weeks post-DMM surgery (5 injections total) at a dosage of 0.5 mg/kg body weight in 20 μl volume using an insulin syringe. **B** The synovial articular cartilage tissues were collected at 8 weeks post-DMM and the efficiency of knockdown of LPCAT3 was analyzed by immunoblotting. Blots represent data from 4 animals/groups. **C** The knee tissues were collected at 8 weeks post DMM and stained with Safranin O fast green (Scale bar- 50 µM). **D**, **E** The sections were graded in a blinded fashion by two independent observers and OARSI score (**D**) and synovitis score (**E**) were presented. **F**-**J** The frozen synovial cartilage tissues were homogenized in the RIPA buffer containing protease inhibitors and indomethacin (1 mg/mL), and centrifuged at 10,000*g* at 4°C to remove tissue debris. The supernatants were collected. The eicosanoids such as PGE_2_ (**F**) and LTB_4_ (**G**) and the cytokines such as TNFα (**H**), IL-6 (**I**), and MCP-1 (**J**) were analyzed by ELISA. Data represent mean ± SD of 2 independent experiments, a total of 8 animals in a group. ***P < .001.*P < .05

The expression of LPCAT3 remains elevated in the DMM-operated mice and correlated with an increase in macrophage infiltration, cytokines, lipid mediators, and cartilage catabolism (Fig. 1). Our in vitro studies revealed that IL-1β promotes cartilage destruction via upregulation of LPCAT3. Therefore, we have examined whether LPCAT3 knockdown would articulatesynovialprotective benefits in mice OA. Previous studies have reported the therapeutic benefits of repeated intraarticular delivery of siRNA in DMM injury-induced OA in mice [41, 46–48]. Based on these studies, we have selected the dose regimen of 5-total injections of LPCAT3 siRNA-lipid nanoparticles (Fig. 6A). Here, we found that intraarticular delivery of LPCAT3 siRNA-lipid nanoparticles suppressed the expression of LPCAT3 by ~ 90% in synovial cartilage compared to scRNA-injected DMM-operated mice (Fig. 6B).The safranin O-fast green stained cartilage sections of scRNA-lipid nanoparticles injected mice showed a marked reduction of the proteoglycans in articular cartilage indicating cartilage damage compared to the sham group (Fig. 6C). The LPCAT3 siRNA liposome therapy significantly protected the loss of articular cartilage (Fig. 6C). The OARSI and synovitis scores were notably higher in the scRNA-injected mice compared to the sham group (Fig. 6D, E & Supplementary Fig. 2B). However, the OARSI score was significantly lower in the LPCAT3 siRNA-injected mice compared to the siRNA group (Fig. 6D, E & Supplementary Fig. 2B). Furthermore, the eicosanoids including PGE_2_ and LTB_4_, and cytokines, TNFα, IL-6, and MCP-1 in the DMM-operated knee tissues were significantly higher in the scRNA injected mice compared to sham-mice (Fig. 6F-J). However, LPCAT3 siRNA-liposome therapy notably suppressed the arachidonic acid-derived eicosanoids (PGE_2_ and LTB_4_) and cytokines such as TNFα, IL-6, and MCP1 in the synovial tissue (Fig. 6F-J).This data suggests that gene-silencing LPCAT3 in the synovial cartilage through the RNAi approach inhibits the generation of cytokines and eicosanoids, and protects degeneration of articular cartilage.

## Discussion

Osteoarthritis is a chronic degenerative disease of the articular cartilage with no curative treatment. The cytokines (TNFα, IL-6) and lipid mediators such as PGE_2_ and LTB_4_ cause arthritic pain, infiltration, and inflammation in the joint tissues [6, 8, 49]. The LPCAT3 plays a crucial role in arachidonic acid metabolism in cells [29, 31]. In this study, we found that the expression of LPCAT3 was upregulated in OA and correlated with the severity of the disease. Here, several lines of evidence using human chondrocytes, human cartilage explants, and in vivo*,* mice models of OA studies consistently showed that the MALT1-LPCAT3 axis promotes cartilage destruction through multiple mechanisms involving upregulation of matrix-degrading enzymes, cytokines, and eicosanoids. The gene silencing of MALT1 or LPCAT3 in chondrocytes blunted the IL-1β-induced secretion of cytokines and eicosanoids and conserved the death of chondrocytes. Local delivery of MALT1 inhibitor in mice manifests similar articular cartilage protective benefits in mice with OA. Most importantly, this study showed that intermittent intraarticular delivery of LPCAT3 siRNA using lipid nanoparticles alleviates secretion of eicosanoids and cytokines, and protects the loss of articular cartilage in the mice model of OA, and human cartilage explants of OA patients. This is the first study that establishes the role of MALT1-LPCAT3 axis in the development of osteoarthritis and provides novel therapeutic avenues targeting this pathway using LPCAT3 siRNA-lipid nanoparticle approach or local injections of MALT1-inhibitors for the treatment of OA.

Accumulated data of evidence suggests that pathological contributions of LPCAT3 were mainly linked to the remodeling of the membrane phospholipids and associated receptor dynamics [30]. In this study, we found that the upregulation of LPCAT3 in OA correlated with the severity of OA. Several lines of evidence in human and animal models of OA suggest that the secretion of eicosanoids contributes to the progression of OA mainly through the infiltration of leukocytes, secretion of cytokines, and upregulation of matrix-degrading enzymes which culminate in cartilage degeneration [50–52]. The PGE_2_ via its cognate EP receptors suppress proteoglycan synthesis and contributes to the pathophysiology of OA [50, 52]. The TNFα and IL-1β promote MMP3 production via PGE_2_signaling [53, 54]. In this study, we found that IL-1β stimulates MMP3 and PGE_2_ production in chondrocytes, and gene silencing of LPCAT3 potentially reduced their synthesis in chondrocytes, human explants, and also in vivo in mice. These multiple lines of evidence support that expression of LPCAT3 in human cartilage contributes to the pathology of OA mainly through the generation of arachidonic acid-derived eicosanoids.

The IL-1β is a potential trigger of osteoarthritis whose levels were elevated in the synovial fluid of the patients and mice models of OA [12, 13, 55]. Our in vivo data was consistent with previous studies where synovial IL-1β levels were significantly elevated in the DMM-injured mice and correlated with the expression of matrix-degrading enzymes and severity of OA. Our in vitro studies revealed that IL-1β treatment triggers the activation of chondrocytes and upregulates the expression of LPCAT3, MMP3, and ADAMTS5. The MALT1 was a scaffolding protein whose function was a downstream signal transducer of many cytokine and antigen receptors [56–58]. Our recent study revealed that MALT1 plays a critical role in the IL-1β-induced cell-signaling and expression of proinflammatory genes in endothelial cells [23]. Therefore, we have examined whether MALT1 plays a role in the IL-1β-induced upregulation of LPCAT3, MMP3, and ADAMTS5 in chondrocytes. Our gene silencing and small-molecule inhibitor approach revealed that the IL-1β-MALT1 axis plays a crucial role in the upregulation of these genes in chondrocytes and cartilage explants. Furthermore, we have overexpressed MALT1to confirm its role in cartilage degeneration. There was significant upregulation of LPCAT3, MMP3, and ADAMTS5 in MALT1-overexpressed chondrocytes. The IL-1β further augmented the expression of the above mentioned genes which confirms that MALT1 plays a critical role in the IL-1β-induced cartilage damage.

The MALT1 is an adaptor protein that integrates the signaling evoked from engaged receptors and activates downstream transcription factors [21, 23, 56, 57]. It has been shown that IL-1β-activates c-Myc and induces inflammatory genes [42–44]. Several studies have shown that c-Myc was involved in the apoptosis of chondrocytes and the pathogenesis of OA [43, 44]. Recent studies demonstrate that MALT1 activity regulates Myc signaling in immune cells [59, 60]. Consistent with these studies, we found that there was a significant accumulation of c-Myc in the MALT1-overexpressed chondrocytes. To assess the role of c-Myc in the IL-1β-MALT1-induced cartilage degeneration, we have incubated the MALT1-overexpressed chondrocytes with specific inhibitors of c-Myc that inhibit its transcriptional activity. We found that inhibition of c-Myc potentially downregulated the expression of LPCAT3, MMP3, and ADAMTS5 in MALT1-overexpressed and/or IL-1β treated chondrocytes. This data indicates that the IL-1β-MALT1-c-Myc pathway was responsible for cartilage degeneration in OA. Corroborating this data, intraarticular therapyof MALT1 specific inhibitor, mepazine, notably inhibited the secretion of cytokines and eicosanoids and conserved the loss of cartilage in DMM-induced OA in mice.

LPCAT3 has been found to play an important role in the development and progression of various diseases underscores it to be a potential therapeutic target [31]. LPCAT3 regulates the expression of C-SRC and TLR4, and promotes the development of atherosclerosis [31]. LPCAT3 regulates other inflammatory factors such as LPC and AA, in vascular smooth muscle, thereby exacerbating atherosclerosis [31]. In addition, LPCAT3 overexpression in skeletal muscle impairs insulin signaling and aggravates glucose intolerance [31]. Consistent with these studies, we found that LPCAT3 expression was upregulated in OA, and correlated with severity of the disease. At present there is no permanent treatment available for OA [61]. Therapeutic approaches include steroids, non-steroidal anti-inflammatory drugs (NSAIDs), IL-6 & TNF-α inhibitors, etc. [62, 63]. These treatments relieve pain and inflammation but do not offer any permanent solution to the OA. The gene silencing by siRNA targeting specific pathways implicated in the pathogenesis of osteoarthritis (OA) is a novel strategy [61]. This approach holds promise and provides new direction for the treatment of OA [61]. Intra articular delivery of siRNA against NF-κB, ADAMTS5, MMP13, and STAT1 alleviated cartilage degeneration in mouse models of OA [39, 46, 61, 64]. Having found the role of LPCAT3 in the progression of OA, we ventured whether targeting LPCAT3 using siRNA-lipocomplex offers protection in OA. LPCAT3 siRNA- lipocomplex therapy abrogated the DMM-induced inflammation and loss of cartilage in mice. Overall, our in vitro and in vivo studies strongly suggest that gene silencing of LPCAT3 provides chondroprotection in OA.

In conclusion, this is the first study that establishes the role of MALT1-LPCAT3 in the development of osteoarthritis. The MALT1-LPCAT3 axis propagates inflammation and cartilage destruction in OA through a multitude of effects of alterations in arachidonic acid, cytokine, and eicosanoid metabolism. Intraarticular administration of LPCAT3 siRNA-lipid nanoparticle complex or MALT1 inhibitors holds therapeutic promise in OA.

## Materials & methods

### Materials

The TNFα and IL-6 ELISA kits, MTT, and apoptosis ELISA detection kit were obtained from Sigma-Aldrich Chemicals (Bangalore, India). The PGE_2_ and LTB_4_ ELISA kits were procured from Enzo Life Sciences (New Delhi, India). The Lipofectamine RNAiMAX reagent, Lipofectamine-2000 reagent, human-specific LPCAT3 siRNA (product ID. 16915), mouse-specific LPCAT3 siRNA (product ID. 89055), human-specific MALT1 siRNA (product ID. 105148) and scrambled siRNAs (product ID. 8390844) were procured from Thermo Fisher Scientific (MA, USA). Mepazine Acetate was purchased from Merck, CA, USA. The inhibitors, KJPyr9, and EN4 were purchased from Sigma-Aldrich Chemicals (Bangalore, India). The LPCAT3 polyclonal antibody was purchased from Novus Biologicals, CO, USA (Product no: NBP3-10074). The human anti-LPCAT3 antibody was purchased from Abcam MA, USA (Product no: ab232958). MALT1 antibody was procured from Cell Signaling Technology, USA (Product no: 2494). Myc (Product no: sc-40) and GAPDH (Product no: sc-32243) antibodies were procured from Santa Cruz Biotechnology, Texas, USA.

### Methods

#### Isolation of chondrocytes

OA human cartilage was obtained from OA patients ages 53–76 years diagnosed according to the International Cartilage Repair Society criteria undergoing total knee replacement. Cartilage samples were cut into small pieces and digested with trypsin–EDTA at 37°C for 20 min. The trypsin solution was washed and tissue slices wereincubated with 2 mg/ml type II collagenase (Sigma Aldrich, Bangalore, India) in DMEM (Himedia Laboratories, Mumbai, India) for 16h. Next, it was filtered using nylon mesh and washed with medium. The isolated chondrocytes were seeded in T75 culture flasks and incubated in DMEM containing 10% fetal bovine serum (FBS), penicillin (100 U/ml), and streptomycin (100 mg/ml) at 37°C in an atmosphere of 5% CO2. At confluence, the cells were detached and seeded in 24-well plates at a density of 1 × 10^5^/well. Cell passages 1 to 2 were used in our study.

#### Transfection of plasmids or siRNA and immunoblotting

Human chondrocytes were seeded in a 24-well plate at a density of 1 × 10^5^cells/well with DMEM and 10%FBS. The next day, transfection of MALT1 plasmids or siRNA was done using Lipofectamine. The chondrocytes were transfected with scrambled RNA (scRNA), LPCAT3 siRNA(200 nM),or MALT1 siRNA (200 nM) using Lipofectamine RNAiMAX transfection reagent (Thermo Fisher, MA, USA) in 500μl RPMI medium for 8 h as described in our previous studies [22, 23, 65]. Then the medium was replaced with serum-richDMEM medium and cultured for 48h or as described in the figure legends. Similarly, 1 μg of pCMV6-XL4-MALT1 or pCMV-XL4 empty plasmids (Origene, MD, USA) were transfected using Lipofectamine 2000 (Thermo Fisher, MA, USA) in DMEM medium without antibiotics for 12h. Later the medium was replaced with fresh medium. 48 h post-transfection, the cells were stimulated with IL-1β as described in figure legends, and cell medium was collected for analysis of cytokines and eicosanoids. Alternatively, the cells were lysed in the SDS-PAGE sample buffer, and an equal amount of protein or volume was subjected to SDS-PAGE, followed by immunoblot analysis. The immunoblots were developed with chemiluminescence using HRP-substrate (Sigma). Densitometric analysis was performed using the Bio-Rad Chemi XRS system and Image J software.

#### RNA extraction and qRT-PCR

Total RNA from the chondrocytes or cartilage tissues was extracted using TRIzol reagent (Sigma Aldrich, Banglore, India). The total RNA was extracted, and cDNA was prepared by reverse transcription (iScript cDNA Synthesis Kit, Bio-Rad) and amplified by PCR (Bio-Rad, CA, USA), as described in our previous studies [65, 66]. The qRT-PCR was done in triplicates and the expression of target genes was normalized to GAPDH. The relative gene expression levels were calculated using the 2^−ΔΔCT^ method and expressed as fold changes [65]. Sequences of the primers used in this study are presented in Supplementary Table 1.

#### Cell viability assay

Cell viability was assessed by MTT assay as described previously [67, 68]. Briefly, human chondrocytes were seeded in 96-well plates at a density of 1.0 × 10^4^ cells/well in DMEM with 10% FBS. The next day, the cells were transfected with scRNA or siRNA targeting LPCAT3 or MALT1 (20 n*M*) for 12h hours in the serum-free medium using Lipofectamine RNAiMAX transfection reagent. Later, the medium was replaced with a complete medium. 48 h post-transfection, the cells were treated with IL-1β for 24 h. In the mepazine treated group, mepazine was added 2h prior toIL-1β treatment. Cell viability was measured by MTT assay. Briefly, fresh MTT (5 mg/ml in PBS) was prepared and 25 μl was added to each well. 4 h later, the supernatant was removed, and 100 μl of DMSO was added to each well. The absorbance of the samples was measured at 540 nm using a plate reader. Cell viability was measured as % absorbance of IL-1β-treated cells compared to the cells not treated with IL-1β.

#### Apoptosis assay by ELISA

The apoptosis assay was determined by a cell-death ELISA detection kit (Sigma-Aldrich, Bangalore, India) that measures histone-DNA complexes generated during DNA fragmentation caused by apoptosis [69, 70]. The assay was done using the manufacturer’s instructions and absorbance was read at 405 nm using a spectrophotometer.

#### Culture of cartilage explants

The Human cartilage explants were cultured as described previously [5]. Briefly, cartilage explants were cut into 4 × 5 mm volumes and they were cultured in DMEM containing 10% FBS. They were transfected with scRNA, human LPCAT3 siRNA, or MALT1 siRNA using Lipofectamine RNAiMax reagent in a serum-free medium for 10 h. Later, the medium was replaced with serum-rich DMEM and 10% FBS with antibiotics. 48 h post-transfection, they were stimulated with IL-1β (10 ng/ml) for 24 h. The cartilage explants were then collected for immunoblot or RT-PCR analysis. The medium was collected for analysis of sulfated glycosaminoglycans (sGAG) by 1, 9, dimethyl methylene blue (DMMB) (Sigma-Aldrich, Bangalore) assay using chondroitin sulfate as standard [71]. The PGE_2_, LTB_4,_ and IL-6 were analyzed in the medium by ELISA.

#### Lipid extraction and fatty acid analysis in the cells

Total lipids in the cells were extracted [25], saponified with 0.5 M KOH and methylated with 40% boron trifluoride in methanol as described in our previous studies [24, 25]. The methyl esters of fatty acids were extracted with hexane. Fatty acid analysis was carried out as described in our previous studies [24, 25]. The arachidonic acid content in the cells was presented as a relative percentage peak area with respect to other fatty acids [24].

#### Synthesis of LPCAT3 siRNA nanoparticles

We synthesized liponanoparticles containing LPCAT3 siRNA, delivered to mice, and assessed its effects on DMM-induced OA. These nanoparticles have been designed for efficient, safe, and targeted transfection of siRNA [72, 73]. The mouse-specific LPCAT3 siRNA (product ID. 89055) and scrambled siRNAs (product ID. 8390844) were procured from Thermo Fisher Scientific. The lipids 1,2-dipalmitoyl-sn-glycero-3-phospho((ethyl-1′,2′,3′-triazole)triethylene glycol) (ammonium salt) (PEG-DPPE), and N-[1-(2,3-Dioleoyloxy)propyl]-N,N,N-trimethylammonium (chloride salt) (DOTAP), cholesterol, lecithin, were purchased from Sigma Aldrich Chemicals, Bangalore, India.

The siRNA encapsulation was performed as described previously [74, 75]. Briefly, the DOTAP, lecithin, cholesterol, and PEG-DPPE were dissolved in ethanol at a molar ratio of 45:15:35:5 to a final concentration of 15 mM. The mice-specific LPCAT3 siRNA or scRNA dissolved at 2 mg/ml in 50 mmol/l citrate, pH 4.0 was added drop-wise to an ethanolic solution of lipid mixture and incubated at 31 °C for 30 min with constant stirring to a final siRNA/lipid weight ratio of 8/1, wt/wt. The ethanol was removed and the neutralization was performed by dialysis against phosphate-buffered saline (PBS), pH 7.4 overnight using a Spectra dialysis membrane. The synthesized LPCAT3-siRNA-lipid nanoparticles were used for mice OA studies.

#### Induction of OA by DMM-surgery in mice knee tissues

The DMM surgery was performed in the hind knee of 10–12 week-old mice following administration of general anesthesia (inhalation of isoflurane in oxygen) by transection of the anterior attachment of the medial meniscotibial ligament in the knee [76].The joint capsule was closed with a continuous 8–0 suture and the subcutaneous layer with 7–0 suture. The skin was closed by tissue adhesive. The skin of the knee joint was resected in the sham-operated group. Later, the buprenorphine (1.0 mg/kg) was given subcutaneously to alleviate pain after surgery.

#### Intraarticular delivery of mepazine or LPCAT3-siRNA nanoparticles

The DMM-operated mice were intraarticularly injected immediately with mepazine or vehicle and thereafter weekly twice post-DMM surgery at a dosage of 10 mg/kg body weight in 20 μl volume using an insulin syringe. The mice were sacrificed at 8-weeks post DMM and knee tissues were collected. The external muscle tissue was removed using scissors and the knee tissues were either fixed in formalin. The LPCAT3-siRNA lipid nanoparticles were administered after DMM surgery in mice, and also at 1, 2, 4, and 6 weeks after surgery (5 doses). The 20 μl of LPCAT3-siRNA lipid nanoparticle was injected IA using a 30-gauge insulin syringe which was equivalent to 0.5 mg/kg body weight of siRNA/mouse. The untreated sham mice received PBS and the scrambled RNA (scRNA) group received non-targeted scrambled RNA. The knee tissues were collected at 8 weeks post DMM and they were fixed in formalin. Alternatively, the synovial articular cartilage tissues were isolated by microdissection using a scapel and frozen immediately for the analysis of cytokines and eicosanoids.

#### Histological analysis of knee tissues

Eight weeks after DMM surgery, the mice were sacrificed and the knee tissues were harvested, fixed in buffered formalin for 72 h, and transferred to 70% alcohol until they were used. The joint tissues were decalcified by immersing them in a decalcifier (stat-lab) for 48 h and then embedded in paraffin for sectioning. The tissues were sectioned (6-μm thick) and selected sections were stained with safranin O-fast green staining [41, 77]. The cartilage damage was measured using the Osteoarthritis Research Society International (OARSI) scoring system [46, 78, 79]. In brief, grade 0 = healthy cartilage; 0.5 = loss of Safranin O staining with no structural changes; 1 = small fibrillation with no loss of cartilage; 2 = vertical clefts down to the layer below the superficial layer; 3–6 = vertical clefts and erosion of calcified cartilage in the articular surface with < 25%, 25%–50%, 50%–75% and > 75%, respectively. The OARSI scoring of cartilage damage was performed by 2 blinded observers. The mean values of scores were used for analysis. The synovitis score (score scale 0–6) was determined based on the thickness of the synovial lining cell layer, and cellular density in the synovial stroma. The mean values of individual scores for both parameters were used for analysis [46, 80, 81]. Briefly, the degree of synovitis in Safranin O stained sections were obtained using a scoring system that measured the thickness of the synovial lining cell layer on a scale of 0–3 (0 = 1–2 cells, 1 = 2–4 cells, 2 = 4–9 cells and 3 = 10 or more cells) and cellular density in the synovial stroma on a scale of 0–3 (0 = normal cellularity, 1 = slightly increased cellularity, 2 = moderately increased cellularity and 3 = greatly increased cellularity).

#### Immunohistochemistry

For immunohistochemistry, the antigen retrieval was done by boiling cartilage tissue sections for 15 min in sodium citrate buffer (10 mM, pH 6.0). The sections were incubated in 3% hydrogen peroxide for 20 min to quench endogenous peroxide activity. After blocking the tissue sections with an antibody diluent containing background reducing components (Thermofisher, MA, USA), they were incubated with control IgG, or primary antibody against LPCAT3 (5 µg/ml) overnight at 4°C. The sections were then incubated with biotin-labeled goat anti-rabbit IgG (1:500) and ultrasensitive streptavidin-HRP (1:500) (Sigma, Bangalore, India) and developed using DAB substrate kit (Sigma-Aldrich, Bangalore, India), mounted, and visualized with an Olympus BX41 microscope.

#### Measurement of cytokines

Chondrocytes were stimulated with IL-1β (10ng/ml) for 24 h or indicated time points as described in the figure legends. The TNFα, IL-6, LTB_4,_ and PGE_2_ levels in the cell supernatants were estimated using ELISA kits according to the manufacturer’s instructions. The synovial cartilage tissues from mice were snap-frozen in liquid nitrogen, and the frozen tissue was pulverized into powder. The powder was suspended in RIPA buffer containing protease inhibitors and indomethacin. The lysate was briefly sonicated and centrifuged at 10,000 × g for 20 min at 4°C. The TNFα, IL-6, MCP1, LTB_4,_ and PGE_2_ levels in tissue extracts were estimated using ELISA kits.

#### Data analysis

All experiments were conducted independently in triplicates. The data were shown as mean ± SD. Normality tests; Shapiro–Wilk was used for normal distribution of data. Most of the data passed the normality test and parametric tests were used to calculate statistical significance. In qRT-PCR experiments, data are expressed as relative fold changes. We analyzed the data for the two groups using unpaired two-tailed *t* test. One-way ANOVA with *post-hoc* Tukey–Kramer test was used to analyze the differences among more than two groups. *p* < 0.05 was considered statistically significant. GraphPad Prism version 8.0.2 was used for statistical analysis.

### Supplementary Information

**Supplementary Material 1.**

## Data Availability

All data were included in this article or supplementary file.

## Abbreviations

OA: Osteoarthritis
LPCAT3: Lysophosphatidylcholine acyltransferase 3
MMPs: Matrix metalloproteinases
AA: Arachidonic acid
PGE_2_: Prostaglandin E_2_
LTB_4_: Leukotriene B_4_
ADAMTS5: A disintegrin and metalloproteinase with thrombospondin motifs-5
Gab2: GRB2 associated binding protein 2
MALT-1: Mucosa-associated lymphoid tissue lymphoma translocation protein 1
NF-κB: Nuclear factor kappa B
RNAi: RNA interference
siRNA: Small interfering RNA
scRNA: Scrambled RNA
COL2A1: Collagen type II alpha 1 chain
c-Myc: Cellular Myc
GAGs: Glycosaminoglycans
PUFA: Polyunsaturated fatty acids
TNFα: Tumor necrosis factor-alpha
EPA: Eicosapentaenoic acid
DHA: Docosahexaenoic acid
IL-1β: Interleukin-1 beta
IFNγ: Interferon-gamma
RIPA Buffer: Radioimmunoprecipitation assay buffer
IL-12: Interleukin-12
IL6: Interleukin-6
ELISA: Enzyme-linked immunosorbent assay
THP1: Human monocytic cell line
HepG2: Hepatoblastoma cell line
LPCAT3: Lysophosphatidylcholine acyltransferase 3
LPS: Lipopolysaccharide
MAPK: Mitogen-activated protein kinase
JNK: Jun N-terminal kinase
DOTAP: N-[1-(2,3-dioleoyloxy)propyl]-N,N,N-trimethylammonium methyl
PCR: Polymerase Chain Reaction

## References

[1] Bijlsma JWJ, Berenbaum F, Lafeber FPJG (2011). Osteoarthritis: an update with relevance for clinical practice. Lancet.

[2] Katz JN, Arant KR, Loeser RF (2021). Diagnosis and treatment of hip and knee osteoarthritis: a review. JAMA.

[3] Wieland HA, Michaelis M, Kirschbaum BJ, Rudolphi KA (2005). Osteoarthritis — an untreatable disease?. Nat Rev Drug Discov.

[4] James SL, Abate D, Abate KH, Abay SM, Abbafati C, Abbasi N, et al. Anonymous, Global, regional, and national incidence, prevalence, and years lived with disability for 354 diseases and injuries for 195 countries and territories, 1990-2017: a systematic analysis for the Global Burden of Disease Study 2017. Lancet. 2018;392:1789–858. 10.1016/S0140-6736(18)32279-7 PMC6227754 30496104

[5] Li R (2022). The proton-activated G protein-coupled receptor GPR4 regulates the development of osteoarthritis via modulating CXCL12/CXCR7 signaling. Cell Death Dis.

[6] Kapoor M, Martel-Pelletier J, Lajeunesse D, Pelletier J-P, Fahmi H (2011). Role of proinflammatory cytokines in the pathophysiology of osteoarthritis. Nat Rev Rheumatol.

[7] Conaghan PG, Cook AD, Hamilton JA, Tak PP (2019). Therapeutic options for targeting inflammatory osteoarthritis pain. Nat Rev Rheumatol.

[8] Laufer S (2003). Role of eicosanoids in structural degradation in osteoarthritis. Curr Opin Rheumatol.

[9] Jayakumar T, SaravanaBhavan P, Sheu JR (2020). Molecular targets of natural products for chondroprotection in destructive joint diseases. Int J Mol Sci.

[10] Blom AB (2007). Crucial role of macrophages in matrix metalloproteinase-mediated cartilage destruction during experimental osteoarthritis: involvement of matrix metalloproteinase 3. Arthritis Rheum.

[11] Glasson SS (2005). Deletion of active ADAMTS5 prevents cartilage degradation in a murine model of osteoarthritis. Nature.

[12] Vincent TL. IL-1 in osteoarthritis: time for a critical review of the literature. F1000Res. 2018;8:934. 10.12688/f1000research.18831.1 PMC6589928 31249675

[13] Farahat MN, Yanni G, Poston R, Panayi GS (1993). Cytokine expression in synovial membranes of patients with rheumatoid arthritis and osteoarthritis. Ann Rheum Dis.

[14] Daheshia M, Yao JQ (2008). The interleukin 1beta pathway in the pathogenesis of osteoarthritis. J Rheumatol.

[15] Goldring MB, Otero M (2011). Inflammation in osteoarthritis. Curr Opin Rheumatol.

[16] Tu C (2019). Schisandrin A Inhibits the IL-1β-induced inflammation and cartilage degradation via suppression of MAPK and NF-κB signal pathways in rat chondrocytes. Front Pharmacol.

[17] Biswas S (2022). Pharmacological inhibition of MALT1 ameliorates autoimmune pathogenesis and can be uncoupled from effects on regulatory T-cells. Front Immunol.

[18] Mc Guire C (2014). Pharmacological inhibition of MALT1 protease activity protects mice in a mouse model of multiple sclerosis. J Neuroinflamm.

[19] Liu W (2016). MALT1 inhibitors prevent the development of DSS-induced experimental colitis in mice via inhibiting NF-κB and NLRP3 inflammasome activation. Oncotarget.

[20] Qin H (2021). MALT-1 inhibition attenuates the inflammatory response of ankylosing spondylitis by targeting NF-κB activation. Injury.

[21] Ferch U (2009). Inhibition of MALT1 protease activity is selectively toxic for activated B cell–like diffuse large B cell lymphoma cells. J Exp Med.

[22] Kondreddy V, Magisetty J, Keshava S, Rao LVM, Pendurthi UR (2021). Gab2 (Grb2-Associated Binder2) plays a crucial role in inflammatory signaling and endothelial dysfunction. Arterioscler Thromb Vasc Biol.

[23] Kondreddy V (2022). The Gab2-MALT1 axis regulates thromboinflammation and deep vein thrombosis. Blood.

[24] Reddy KVK, Naidu KA (2016). Oleic acid, hydroxytyrosol and n-3 fatty acids collectively modulate colitis through reduction of oxidative stress and IL-8 synthesis; in vitro and in vivo studies. Int Immunopharmacol.

[25] Kondreddy VKR, Anikisetty M, Naidu KA (2016). Medium-chain triglycerides and monounsaturated fatty acids potentiate the beneficial effects of fish oil on selected cardiovascular risk factors in rats. J Nutr Biochem.

[26] Kondreddy VK, Kamatham AN (2016). Celecoxib, a COX-2 inhibitor, synergistically potentiates the anti-inflammatory activity of docosahexaenoic acid in macrophage cell line. Immunopharmacol Immunotoxicol.

[27] Reddy KV, Maheswaraiah A, Naidu KA (2014). Rice bran oil and n-3 fatty acid-rich garden cress (Lepidium sativum) seed oil attenuate murine model of ulcerative colitis. Int J Colorectal Dis.

[28] Fujishima H (1999). Cytosolic phospholipase A<sub>2</sub> is essential for both the immediate and the delayed phases of eicosanoid generation in mouse bone marrow-derived mast cells. Proc Natl Acad Sci USA.

[29] Hashidate-Yoshida T (2015). Fatty acid remodeling by LPCAT3 enriches arachidonate in phospholipid membranes and regulates triglyceride transport. Elife.

[30] Ferrara PJ (2021). Lysophospholipid acylation modulates plasma membrane lipid organization and insulin sensitivity in skeletal muscle. J Clin Invest.

[31] Shao G (2022). Research progress in the role and mechanism of LPCAT3 in metabolic related diseases and cancer. J Cancer.

[32] Zhang H (2022). The regulation of LPCAT3 by miR-124–3p.1 in acute kidney injury suppresses cell proliferation by disrupting phospholipid metabolism. Biochem Biophys Res Commun.

[33] Liu N (2020). Hyperuricemia induces lipid disturbances mediated by LPCAT3 upregulation in the liver. FASEB J.

[34] Ke P (2022). LPCAT3 is a potential prognostic biomarker and may be correlated with immune infiltration and ferroptosis in acute myeloid leukemia: a pan-cancer analysis. Transl Cancer Res.

[35] Guo Z (2022). Deferoxamine alleviates osteoarthritis by Inhibiting chondrocyte ferroptosis and activating the Nrf2 pathway. Front Pharmacol.

[36] Dana H (2017). Molecular mechanisms and biological functions of siRNA. Int J Biomed Sci.

[37] Wang J, Lu Z, Wientjes MG, Au JL (2010). Delivery of siRNA therapeutics: barriers and carriers. Aaps j.

[38] Schroeder A, Levins CG, Cortez C, Langer R, Anderson DG (2010). Lipid-based nanotherapeutics for siRNA delivery. J Intern Med.

[39] Rai MF (2019). Applications of RNA interference in the treatment of arthritis. Transl Res.

[40] Lee SJ (2014). TNF-α gene silencing using polymerized siRNA/thiolated glycol chitosan nanoparticles for rheumatoid arthritis. Mol Ther.

[41] Yan H (2016). Suppression of NF-κB activity via nanoparticle-based siRNA delivery alters early cartilage responses to injury. Proc Natl Acad Sci.

[42] Lemos DR (2018). Interleukin-1β activates a MYC-dependent metabolic switch in kidney stromal cells necessary for progressive tubulointerstitial fibrosis. J Am Soc Nephrol.

[43] Liu G (2022). Phenformin down-regulates c-Myc expression to suppress the expression of pro-inflammatory cytokines in keratinocytes. Cells.

[44] Zou J, Li XL, Shi ZM, Xue JF (2018). Effects of C-myc gene silencing on interleukin-1β-induced rat chondrocyte cell proliferation, apoptosis and cytokine expression. J Bone Miner Metab.

[45] Yatsugi N (2000). Apoptosis of articular chondrocytes in rheumatoid arthritis and osteoarthritis: correlation of apoptosis with degree of cartilage destruction and expression of apoptosis-related proteins of p53 and c-myc. J Orthop Sci.

[46] Duan X (2021). Amelioration of posttraumatic osteoarthritis in mice using intraarticular silencing of periostin via nanoparticle-based small interfering RNA. Arthritis Rheumatol.

[47] Akagi R (2014). Effective knock down of matrix metalloproteinase-13 by an intra-articular injection of small interfering RNA (siRNA) in a murine surgically-induced osteoarthritis model. J Orthop Res.

[48] Tong W (2019). Wnt16 attenuates osteoarthritis progression through a PCP/JNK-mTORC1-PTHrP cascade. Ann Rheum Dis.

[49] Sanchez-Lopez E, Coras R, Torres A, Lane NE, Guma M (2022). Synovial inflammation in osteoarthritis progression. Nat Rev Rheumatol.

[50] Li X (2009). Prostaglandin E2 and its cognate EP receptors control human adult articular cartilage homeostasis and are linked to the pathophysiology of osteoarthritis. Arthritis Rheum.

[51] Gosset M (2008). Mechanical stress and prostaglandin E2 synthesis in cartilage. Biorheology.

[52] Attur M (2008). Prostaglandin E2 exerts catabolic effects in osteoarthritis cartilage: evidence for signaling via the EP4 receptor1. J Immunol.

[53] Sanchavanakit N, Saengtong W, Manokawinchoke J, Pavasant P (2015). TNF-α stimulates MMP-3 production via PGE2 signalling through the NF-kB and p38 MAPK pathway in a murine cementoblast cell line. Arch Oral Biol.

[54] Ruwanpura SM, Noguchi K, Ishikawa I (2004). Prostaglandin E2 regulates interleukin-1beta-induced matrix metalloproteinase-3 production in human gingival fibroblasts. J Dent Res.

[55] Požgan U (2010). Expression and activity profiling of selected cysteine cathepsins and matrix metalloproteinases in synovial fluids from patients with rheumatoid arthritis and osteoarthritis. Biol Chem.

[56] Gross O (2008). Multiple ITAM-coupled NK-cell receptors engage the Bcl10/Malt1 complex via Carma1 for NF-κB and MAPK activation to selectively control cytokine production. Blood.

[57] Lin X, Wang D (2004). The roles of CARMA1, Bcl10, and MALT1 in antigen receptor signaling. Semin Immunol.

[58] Demeyer A, Staal J, Beyaert R (2016). Targeting MALT1 proteolytic activity in immunity, inflammation and disease: good or bad?. Trends Mol Med.

[59] Dai B (2017). B-cell receptor-driven MALT1 activity regulates MYC signaling in mantle cell lymphoma. Blood.

[60] Rosenbaum M (2022). MALT1 protease function in regulatory T cells induces MYC activity to promote mitochondrial function and cellular expansion. Eur J Immunol.

[61] Kumari A, Kaur A, Aggarwal G (2023). The emerging potential of siRNA nanotherapeutics in treatment of arthritis. Asian J Pharm Sci.

[62] Cho Y (2021). Disease-modifying therapeutic strategies in osteoarthritis: current status and future directions. Exp Mol Med.

[63] Apparailly F, Jorgensen C (2013). siRNA-based therapeutic approaches for rheumatic diseases. Nat Rev Rheumatol.

[64] Chen LX (2008). Suppression of early experimental osteoarthritis by in vivo delivery of the adenoviral vector-mediated NF-κBp65-specific siRNA. Osteoarthritis Cartilage.

[65] Kondreddy V (2018). Factor VIIa induces anti-inflammatory signaling via EPCR and PAR1. Blood.

[66] Magisetty J, Gadiraju B, Kondreddy V (2023). Genomic analysis in the colon tissues of omega-3 fatty acid-treated rats identifies novel gene signatures implicated in ulcerative colitis. Int J Biol Macromol.

[67] Ma G (2021). Blockade of TRPM7 alleviates chondrocyte apoptosis and articular cartilage damage in the adjuvant arthritis rat model through regulation of the Indian Hedgehog signaling pathway. Front Pharmacol.

[68] Akasaki Y (2014). FoxO transcription factors support oxidative stress resistance in human chondrocytes. Arthritis Rheumatol.

[69] Lee HG, Yang JH (2012). PCB126 induces apoptosis of chondrocytes via ROS-dependent pathways. Osteoarthritis Cartilage.

[70] López-Armada MJ (2006). Cytokines, tumor necrosis factor-α and interleukin-1β, differentially regulate apoptosis in osteoarthritis cultured human chondrocytes. Osteoarthritis Cartilage.

[71] Bougault C (2012). Stress-induced cartilage degradation does not depend on the NLRP3 inflammasome in human osteoarthritis and mouse models. Arthritis Rheum.

[72] Kong F, Zhou F, Ge L, Liu X, Wang Y (2012). Mannosylated liposomes for targeted gene delivery. Int J Nanomedicine.

[73] Chen P (2014). Optimal structural design of mannosylated nanocarriers for macrophage targeting. J Control Release.

[74] Kulkarni JA (2018). On the formation and morphology of lipid nanoparticles containing ionizable cationic lipids and siRNA. ACS Nano.

[75] Basha G (2011). Influence of cationic lipid composition on gene silencing properties of lipid nanoparticle formulations of siRNA in antigen-presenting cells. Mol Ther.

[76] Glasson SS, Blanchet TJ, Morris EA (2007). The surgical destabilization of the medial meniscus (DMM) model of osteoarthritis in the 129/SvEv mouse. Osteoarthritis Cartilage.

[77] Wang C (2020). Metformin mitigates cartilage degradation by activating AMPK/SIRT1-mediated autophagy in a mouse osteoarthritis model. Front Pharmacol.

[78] Bannuru RR (2019). OARSI guidelines for the non-surgical management of knee, hip, and polyarticular osteoarthritis. Osteoarthritis Cartilage.

[79] Gerwin N, Bendele AM, Glasson S, Carlson CS (2010). The OARSI histopathology initiative - recommendations for histological assessments of osteoarthritis in the rat. Osteoarthritis Cartilage.

[80] Lewis JS (2011). Acute joint pathology and synovial inflammation is associated with increased intra-articular fracture severity in the mouse knee. Osteoarthritis Cartilage.

[81] Zhang Z (2016). Curcumin slows osteoarthritis progression and relieves osteoarthritis-associated pain symptoms in a post-traumatic osteoarthritis mouse model. Arthritis Res Ther.

