# Supplementary material for: Inhibition of the MALT1-LPCAT3 axis protects cartilage degeneration and osteoarthritis
Source: Cell Commun Signal. 2024 Mar 22;22:189. doi: 10.1186/s12964-024-01547-4 (PMC10960471; doi:10.1186/s12964-024-01547-4)
Supplement: Supplementary file 1 — Supplementary Material 1. [file 12964_2024_1547_MOESM1_ESM.docx]

**Inhibition of MALT1-LPCAT3 axis protects cartilage degeneration and osteoarthritis**

Vijay Kondreddy*^1^, Rajkumar Banerjee^1^, Prabhavathi Devi BLA^1^, Kathirvel Muralidharan^2^, Selvakumar Piramanayagam.^2^

^1^Department of Lipid Science and Technology, ^2^Division of Applied Biology, The Indian Institute of Chemical Technology, Tarnaka, Hyderabad, India

Running title: MALT1-LPCAT3 and osteoarthritis

Corresponding author’s contact information:

*Vijay Kondreddy

^1^Department of Lipid Science and Technology

The Indian Institute of Chemical Technology,

Uppal Road, Tarnaka, Hyderabad, 500007, India

Email: [vijay.667@csiriict.in](mailto:vijay.667@csiriict.in)

Phone: 040-27193179

**Supplementary Material**

**Supplementary Table 1: List of qPCR primers used in this study**

| **Gene** | **Forward primer (5^1^-3^1^)** | **Reverse primer(5^1^-3^1^)** |
| --- | --- | --- |
| *hLpcat3* | CCT ACC TCA TCC ACC TCT TC | AGT CAA CAG CCA AAC CAA TC |
| *hMmp3* | AAT GGA CAA AGG ATA CAA CAG | GTG AGT GAG TGA TAG AGT GGG |
| *hCol2A1* | CCC TCA AGG ATT TCA AGG C | AAC CAC TCT CAC CCT TCA C |
| *hAdamts5* | ATC TTT TCT TTC CCC CCC C | ACA CAC ACA CAC TTG CTT G |
| *hIL-6* | AGA AAA CAA CCT GAA CCT TCC | ATA CCT CAA ACT CCA AAA GAC |
| *hMALT1* | AAGCCCTATTCCTCACTACC | CACTGCCTCATCTGTTCTTC |
| *hc-Myc* | TCTTCCCCTACCCTCTCAAC | TGCCTCTTTTCCACAGAAAC |

**Supplementary Figure I: Representative uncropped immunoblot images of each antibody that correspond to cropped images shown in various figures as indicated in parenthesis) of the main manuscript. (A)** GAPDH (Figure 3N) **(B) MALT1** (Figure 3A) **(C) LPCAT3** (Figure 2A)

**(D)** GAPDH (Figure 2I)

**
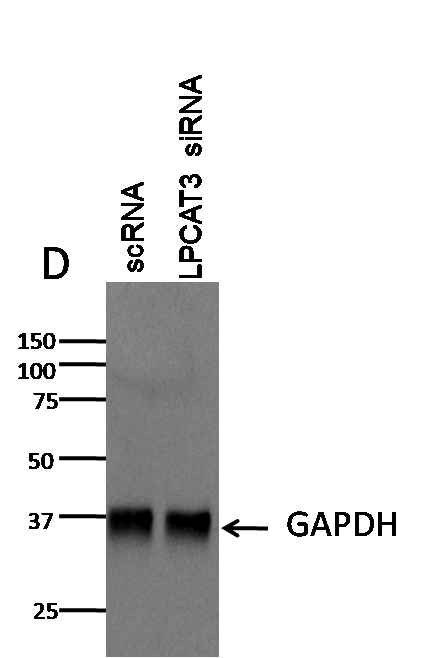

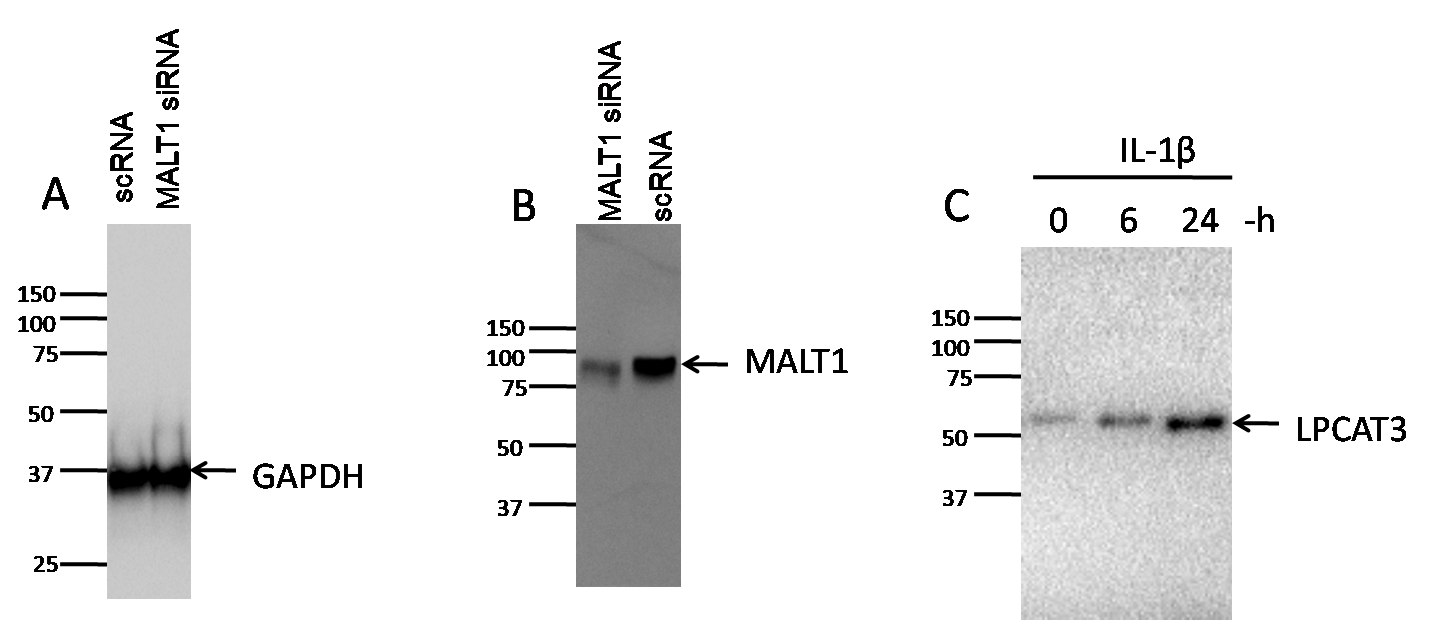
**


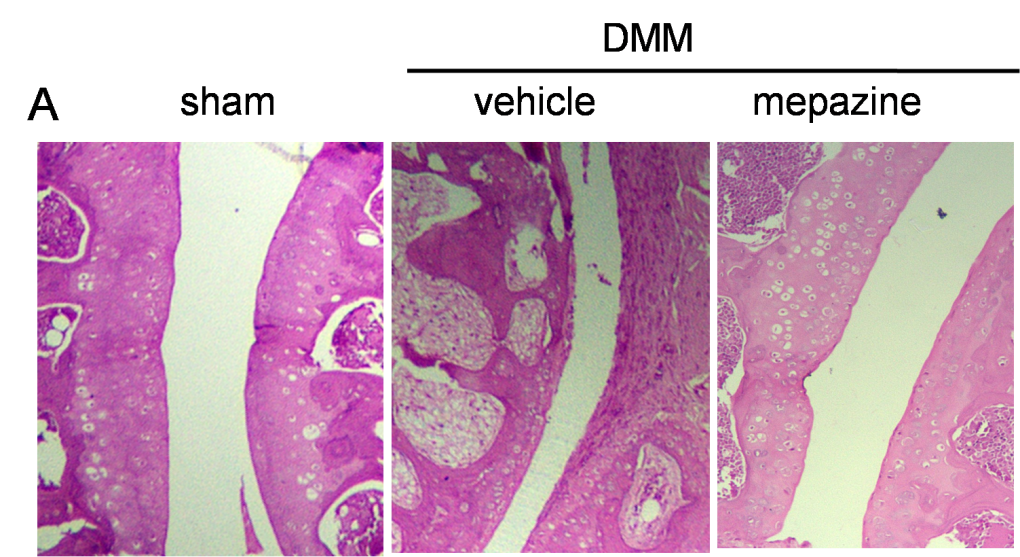


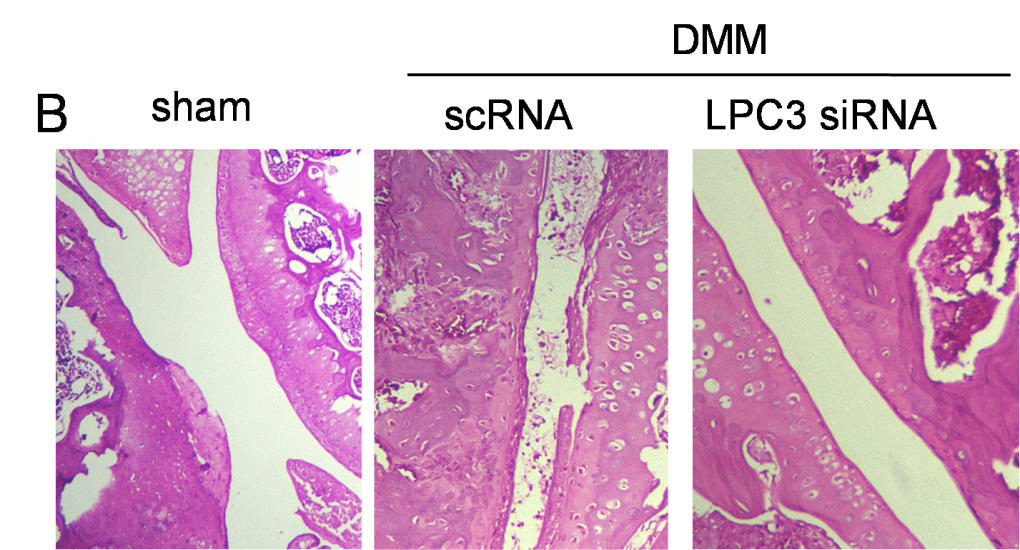


**Supplementary Figure 2: Representative hematoxylin and eosin stained images of knee cartilage sections of mice treated with either mepazine or LPCAT3 siRNA liposomes.**

**(A,B)** OA was induced in knee tissues of mice by performing DMM surgery in 10-12-week-old C57WT mice, followed by intraarticular injection of mepazine **(A)** (n = 8 mice per group), administered immediately and thereafter weekly twice post-DMM surgery. The knee tissues were collected at 8-weeks post DMM and they were fixed in formalin. The formalin fixed sections were decalcified and stained with hematoxylin and eosin. **(B)** OA was induced in knee tissues of mice by performing DMM surgery in 10-12 week-old C57WT mice, followed by intraarticular injection of LPCAT3 siRNA lipid nanoparticles (LPC3 siRNA) or scrambled siRNA (scRNA) or PBS as an untreated control (sham), administered immediately and thereafter at 1, 2, 4, and 6 weeks post-DMM surgery (5 injections total) at a dosage of 0.5 mg/kg body weight in 20 μl volume using an insulin syringe. The knee tissues were collected at 8-weeks post DMM and stained with hematoxylin and eosin. The sections were graded for synovitis score by two blinded observers according to the protocol described in the methodology section. The images represent the data of synovitis score presented in main Figures 5C and 6E.
